# Effect of pomegranate extracts on brain antioxidant markers and cholinesterase activity in high fat-high fructose diet induced obesity in rat model

**DOI:** 10.1186/s12906-017-1842-9

**Published:** 2017-06-27

**Authors:** Zahra Amri, Asma Ghorbel, Mouna Turki, Férièle Messadi Akrout, Fatma Ayadi, Abdelfateh Elfeki, Mohamed Hammami

**Affiliations:** 1 Biochemistry Laboratory, LR12ES05 “Nutrition- Functional Foods and vascular Health”, Faculty of Medicine, University of Monastir, 5019 Monastir, Tunisia; 2 Laboratoire d’Hygiène CHU Hédi Chaker, Sfax, Tunisia; 3 Biochemistry Laboratory, CHU H. Bourguiba, Sfax, Tunisia; 4 Laboratory of Animal Ecophysiology, Faculty of Sciences of Sfax, University of Sfax, Sfax, Tunisia

**Keywords:** Obesity, Brain, Pomegranate, Oxidative stress, Anti-cholinesterase activity

## Abstract

**Background:**

To investigate beneficial effects of Pomegranate seeds oil (PSO), leaves (PL), juice (PJ) and (PP) on brain cholinesterase activity, brain oxidative stress and lipid profile in high-fat-high fructose diet (HFD) induced-obese rat.

**Methods:**

In vitro and in vivo cholinesterase activity, brain oxidative status, body and brain weight and plasma lipid profile were measured in control rats, HFD-fed rats and HFD-fed rats treated by PSO, PL, PJ and PP.

**Results:**

In vitro study showed that PSO, PL, PP, PJ inhibited cholinesterase activity in dose dependant manner. PL extract displayed the highest inhibitory activity by IC50 of 151.85 mg/ml. For in vivo study, HFD regime induced a significant increase of cholinesterase activity in brain by 17.4% as compared to normal rats. However, the administration of PSO, PL, PJ and PP to HDF-rats decreased cholinesterase activity in brain respectively by 15.48%, 6.4%, 20% and 18.7% as compared to untreated HFD-rats. Moreover, HFD regime caused significant increase in brain stress, brain and body weight, and lipid profile disorders in blood. Furthermore, PSO, PL, PJ and PP modulated lipid profile in blood and prevented accumulation of lipid in brain and body evidenced by the decrease of their weights as compared to untreated HFD-rats. In addition administration of these extract protected brain from stress oxidant, evidenced by the decrease of malondialdehyde (MDA) and Protein carbonylation (PC) levels and the increase in superoxide dismutase (SOD) and glutathione peroxidase (GPx) levels.

**Conclusion:**

These findings highlight the neuroprotective effects of pomegranate extracts and one of mechanisms is the inhibition of cholinesterase and the stimulation of antioxidant capacity.

## Introduction

Obesity is widely known as risk factor for diabetes [1], cardiovascular diseases and neurodegenerative diseases [2–4] such as Alzheimer’s disease (AD) which is a the most prevalent form of dementia characterized by a progressive decline in memory, behavior, and cognitive functions in the elderly population [5]. Based on current data, regression studies have shown that higher body mass index (BMI) was associated with decreased brain volume [6, 7] and people with higher midlife BMI or central adiposity measures have a two times higher risk of dementia in later life [8–10]. In addition, clinical studies have reported deficits in learning, memory and executive function in obese as compared to non-obese patients [11] and have confirmed alterations and reduction in focal gray matter volume in obese young adults [12]. Moreover, experimental studies have shown that obesity altered brain structure and function and which lead to cognitive deficit [13, 14]. Indeed, cognitive decline was correlated with degeneration of cholinergic system [15] caused by increasing in cholinesterase activity. Bon fleur et al. 2000 have demonstrated that AchE activity was significantly higher in cortex, hypothalamus and total brain samples in monosodium L-glutamate (MSG)—induced obese mice and rats as compared to control [16]. Also Pistell et al. 2010 have reported that High Fat Lard diet increased body weight, impaired cognition and increased brain inflammation [6]. Furthermore, Zhang et al. 2005 have explained that increased oxidative stress as mediator of cells death induces neural oxidative stress and inflammation in rat cerebral cortex [17]. The evident correlation between obesity and dementia prompted researchers to look for healthy diet with high antioxidant impact given that long term use of neurodegenerative diseases drugs can induce adverse side effects that may not be tolerated by patients [18].

More recently, the interest in the role of dietary antioxidants in human health has prompted research in the field of AD [19–21]. In this regard, pomegranate, considered recently as functional food exhibited a variety of healthy effects [22]. In fact, different pomegranate parts have been known as an arsenal of bioactive compounds such as phenolics, anthocyanins, vitamins and minerals [23, 24]. This richness attributed to them a power antioxidant activity and protective effects against major chronic diseases such as obesity, diabetes, cancer and Alzheimer [1, 25–28]. Several studies have investigated the protective effect of pomegranate parts on different organs such as liver [29–31], heart [32] and skeletal muscle [33] in obese rat model. But till now, there are no studies conducted to find out the effect of long-term pomegranate consumption on oxidative status in brain in obese rat. To fill the information gap, we designed this study to investigate whether consumption of pomegranate parts: oil seeds (PSO), juice (PJ), peel (PP) and leaves (PL) would alleviate oxidative status and cognitive decline in HFD-induced obesity rat model.

## Methods

### Plant materials

Pomegranate leaves and fruits were harvested from Tounsi trees in October 2015 from Mahdia region, Tunisia. Variety authenticity was confirmed by taxonomist Dr. Faten Zaouay from the Department of Horticulture, Higher Agronomic Institute (University of Sousse, Tunisia) and a voucher specimen was deposited in herbarium at the Faculty of Pharmacy (University of Monastir, Tunisia).

Fruits were washed and hand-peeled. Arils were squeezed using a commercial blender (moulinex, France). The extract juice was centrifuged at 15000 rpm for 15 min. Then the supernatant was recuperated and lyophilized.

The grains were manually separated from the pulp, carefully washed and dried in the sun until constant weight. Then, the grains were crushed and sieved to obtain fine powders. Leaves and peel were sundried and powdered.

### Extraction

Leaves and peel powders were extracted with methanol 50 g/250 ml in the dark for 48 h. Each extract was filtered and evaporated to dryness and stored at −20 °C for further determination.

Oil was extracted from pomegranate seeds by the methods of soxhlet. About 30 g seeds were extracted with 200 ml of hexane at room temperature for 6 h. The solvent was removed by evaporation at 40 °C and the oil was flushed with nitrogen stream and stored at −20 °C in sealed tubes.

### In vitro determination of AChE activity

The AchE assay was performed according to the colorimetric methods of Ellman et al. [34, 35], with some modifications using as a substrate the propionylthiocholine iodide (PTCI), the commonly used substrate for in vitro AChE determinations. Heparinized plasma was prepared from human blood volunteers to use as enzyme source. 100 μl of each pomegranate extracts was mixed with an enzyme solution (100 μl) and incubated at 37 °C for 10 mn. Absorbance at 405 nm was read immediately after adding an Ellman’s reaction mixture 0.25 mM 5.5 5, 5′-Dithiobis 2-nitrobenzoic acid (DTNB); 7 mM PTCI; 50 mM sodium phosphate buffer (pH 7.7). The blank reaction was measured by substituting saline for the enzyme. The percentage of AchE inhibition was calculated using the following formula (1-S/E) × 100; where E and S were the enzyme activities without and with the test sample, respectively. IC50 values were determined from the inhibition percentage values of different concentrations of each plant extract tested in triplicate.

### Experimental design

35 healthy adult male *Wistar* rats weighing about 200–250 g were purchased from the central pharmacy of Tunisia (SIPHAT, Tunis City, Tunisia). At arrival, animals were acclimatized for a period of 7 days in environmentally controlled breeding room standard environmental conditions such controlled temperature (22 ± 2 °C), a 12-h light/dark cycle and a humidity of 60 ± 5. Experimental procedures were approved by Animal Ethics Committee of the University of Sfax (Sfax, Tunisia) for the care and use of laboratory animals.

Animals were divided into 6 groups of 6 animals each and allowed free access to tap water and alimentation during the experimental period. Standard diet was composed by corn, soya and vitamins. High fat-high fructose diet was composed by 69.9% standard diet, 15% animal fat, 15% fructose and 0.1% cholic acid. The experimental groups were as followed:

- Control group (CG): rats fed a standard diet and received daily 1 ml of tap water by gavage
- HFD group (HFD): rats fed a high fat-high fructose diet and received daily 1 ml of tap water by gavage
- HFD+ PSO group (HFD+ PSO): rats fed with a high fat-high fructose diet and received pomegranate seeds oil (2 ml/kg of BW daily)
- HFD+ PJ group (HFD+ PJ): rats fed with a high fat-high fructose diet and received pomegranate juice (250 mg/kg of BW daily)
- HFD+ PP group (HFD+ PP): rats fed with a high fat-high fructose diet and received pomegranate peel (250 mg/kg of BW daily)
- HFD+ PL group (HFD+ PL): rats fed with a high fat-high fructose diet and received pomegranate leaves (250 mg/kg of BW daily)

At the end of treatment period (12 weeks), rats were killed by decapitation. Blood was collected and plasma was processed for lipid profile, cholinesterase activity and oxidative stress assessment. Whole brain was carefully dissected, rapidly removed, weighed and homogenized in phosphate buffer pH 7.4 with an ultrathurax homogenizator. After centrifugation at 10000 g, 4 °C for 10 min, supernatant was used for determination of brain enzymes activities: BchE and oxidative stress.

### Determination of ChE inhibitory activity in brain

The BChE inhibitory activity was performed according to the colorimetric method, using butyrylthiocholine as a substrate (CHRONOLAB system). Protein concentration was determined according to the lowry method with bovine serum albumin (BSA), as a protein standard. The rates of hydrolysis by BChE were monitored spectrophotometrically. 1.5 ml of substrate was mixed with 10 μl of enzyme solution (brain homogenate or plasma samples). The yellow reaction product was quantified by reading immediately the absorbance at 405 nm. The reaction was monitored over a period of 1.5 min with readouts taken every 30s. Quantification of the enzymatic activity was based on a change in optical density in the linear range over time, using the molar extinction coefficient of the reaction products. The spectrophotometric absorption was quantitatively measured and expressed as mole of butyrylcholine hydrolysed/mn/ml plasma or /g cortical tissue protein.

### Biochemical plasma analysis

The levels of total cholesterol (TC), total triglycerides (TG), low density lipoprotein cholesterol (LDL-C) and high density lipoprotein- cholesterol (HDL-C), as well as the activities of Alanine Aminotransferase (ALT) and aspartate aminotransferase (AST) were determined in fasting blood using commercially available kits (Bekman Counter, Galway, Ireland).

### Evaluation of brain antioxidant status

#### Determination of malondialdehyde

Brain lipoperoxidation was evaluated by malondialdehyde (MDA) measurement using thiobarbituric acid reactive substances (TBARS) assay according to the method of [36]. Spectrophotometric measurement was done at 532 nm and the results were expressed as the amount of nmole of MDA per mg of protein.

#### Determination of protein carbonylation

Oxidative damage to proteins was evaluated by quantifying protein carbonylation quantification in brain homogenate according to [37]. Homogenate was incubated with 2, 4, dinitrophenylhydrazine 10 mM DNPH containing buffer for 1 h at room temperature and then proteins were precipitate with 20% (*w*/*v*) trichloroacetic acid (TCA). After centrifugation at 4000 rpm for 5 mn, the supernatant was removed and the precipitate was washed firstly with 10% (*w*/*v*) TCA solution and then by of 1:1 (*v*/v) ethanol: ethyl acetate and finally mixed with 6 M guanidine hydrophilic solution. After vigorous vortex, the mixture was centrifuged at 4000 rpm and the absorbance of the supernatant was measured at 370 nm. The protein carbonyl content was expressed as μM.

#### Determination of superoxide dismutase (SOD)

Superoxide dismutase (SOD) activity was determined according to the method of [38]. Enzymatic activity is directly proportional to the inhibition rate of nitroblue tetrazolium (NBT) oxidation by O_2_^−^ anion. The absorbance was measured at 580 nm and the activity was expressed as U/mg protein in liver and kidneys. One unit of SOD activity was defined as the amount of enzyme that inhibits 50% of NBT reduction.

#### Determination of glutathione peroxidase (GPx)

Glutathione peroxidase (GPx) activity was measured as described by Flohe & Gunzler [39]. Glutathione (GSH) oxidation by GPx is coupled to the transformation of 5, 50-dithiobis-(2- nitrobenzoic acid) (DTNB) into 2-nitro-5-thiobenzoate (TNB) which absorbs at 412 nm. Enzymatic activity was expressed as U/g protein in liver and kidneys.

### Statistical analysis

Results are expressed as means ± SEM (Standard Error of the Mean) and analyzed using SPSS ver. 21.0, professional edition. A one-way analysis of variance (ANOVA) was then performed and followed by Duncan’s test to estimate the significance among the main effects at the 5% probability level. Significant differences (*P* < 0.05) between means were identified by multiple comparisons across the six groups using least significant difference (LSD) procedures.

## Results

### Polyphenol contents of pomegranate extracts

The total phenols, Tanins, flavonoids, anthocyanins and total carotenoids content of pomegranate leaf, Juice and peel extracts in *Tounsi* variety were quantified. Results showed that Peel extract exhibited the highest content of phenols (382 ± 6.36 mg GAE/g DW), carotenoids (57.13 ± 10.84 mg/g DW) and tannins (68.33 ± 0.87 mg tannic acid/ g DW). Leaves extract showed the highest amount of flavonoids (54.84 ± 0.58 mg catechin/g DW) and Juice extract was more concentrated in anthocyanins (441.48 ± 12.03 mg/g DW).

### Effect of PSO, PL, PJ and PP on AchE activity in vitro

The ability of different pomegranate extracts to inhibit AchE was evaluated by the widely accepted modified Ellman’s method and the results are summarized in the Table 1.

**Table 1:** IC50 values of AChE inhibitory activity in vitro of pomegranate extracts

	PSO	PJ	PP	PL
IC50	229.38 ± 2.66^b^	181.52 ± 8.56^ab^	349.31 ± 57.95^c^	151.85 ± 7.42^a^

Values represent mean ± SD of replicate experiments (*n* = 3). (*p* < 0.05)

All the tested extracts inhibited the AchE in dose dependant manner. The comparison between IC50 values of different extracts revealed that leaves extracts displayed the highest inhibitory activity against AchE and it exhibited the lower IC50 value of 151.85 mg/ml. Pomegranate juice was found to be the second stronger inhibitor with IC50 value of 181.52 mg/ml followed by PSO, which exhibited moderate AchE inhibition. However, pomegranate peel appears as the weakest inhibitor. The anti-cholinesterase activity of some pomegranate extracts was reported by previous studies [40, 41] but to the best of our knowledge, this is the first comparative study between different pomegranate parts on anti-cholinesterase activity.

### Effect of PSO, PL, PJ and PP on on BchE activity in brain of HFD-rats

**Fig. 1:**
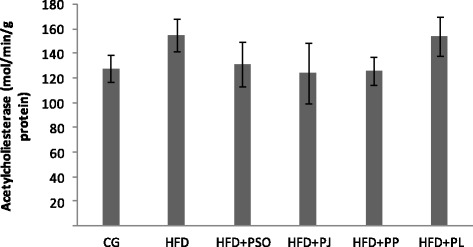
Effect of different pomegranate parts on activity of Butyrylcholinesterase in HFD rat brain. Pomegranate seeds oil (PSO). Pomegranate Juice (PJ). Pomegranate Peel (PP) and Pomegranate Leaves (PL). No significant difference was recorded between all groups

In order to determine effect of HFD regime and pomegranate consumption on BchE level in vivo, brain homogenate was assayed using the colorimetric method. As illustrated in Fig. 1, results showed that HFD regime increased BchE level in rat brain by 17.4% compared to control group. Administration of PSO, PL, PJ and PP to HDF-rats reduced brain BchE level respectively by 15.48%, 6.4%, 20% and 18.7% as compared to untreated HFD-rats but this reduction was not statistically significant.

### Effect of PSO, PL, PJ and PP on body and brain weights of HFD-rat

Data in the Table 2 demonstrated that HFD diet induced a significant increase of body and brain weights respectively by 14.42% and 10.42% as compared to normal rats. However, the administration of PSO, PL, PJ and PP to HFD-rats decreased body weight respectively by 6%, 10.44%, 7% and 9.4% and brain weight by 4.29%, 8%, 9.2% and 7.9% as compared to untreated HFD-rats.

**Table 2:** Effects of pomegranate extracts on body and brain weight

	CG	HFD	HFD + PSO	HFD + PJ	HFD + PP	HFD + PL
Start weight	240.8 ± 4.62	236 ± 7.03	236.8 ± 10.57	239.6 ± 4.02	237.4 ± 6.24	237.8 ± 8.7
End weight	352.4 ± 10.71	411.8 ± 6.01^a^	387 ± 5.56^ab^	383 ± 11.85^ab^	372.8 ± 4.6^ab^	368.8 ± 17.23^ab^
Brain weight	1.46 ± 0.04	1.63 ± 0.07^a^	1.56 ± 0.06	1.48 ± 0.12^b^	1.50 ± 0.07^b^	1.5 ± 0.02

Data are expressed as mean ± SEM (*n* = 6); ^a^ *p* < 0.05 vs control group; ^b^ *p* < 0.05 vs HFD group

Brain weight gain was corrected significantly only by pomegranate juice and pomegranate peel extract. However no corrective effect was observed in PSO and PL treated groups. So it is clear that HFD regime doesn’t induce brain atrophy. Same finding was found in previous study about impact of grape seeds and skin extracts on brain lipotoxicity in rats fed HFD. In fact, they remarked that HFD increased brain weight and induced cholesterol and phospholipids accumulation [42].

### Effect of PSO, PL, PJ and PP on lipid plasma profile and liver enzymes

Results presented in Table 3 shows that HFD regime contributed to a significant elevation estimated by 50%, 72% and 62% in respective lipid plasma parameters CT, LDL-C and TG and a significant decrease in HDL-C level by 42%, compared HFD groups to control groups. This result was confirmed by almost HFD regime studies which concluded that such regime clearly induced obesity characterized by body weight gain, disturbed plasma triglyceride, cholesterol, phospholipid, and abdominal fat accumulation [42].

**Table 3:** Effects of pomegranate extracts on plasma lipid profile and liver enzyme content

Groups	GC	HFD	HFD + PSO	HFD + PJ	HFD + PP	HFD + PL
TC (mmol/l)	1.64 ± 0.25	3.28 ± 0.33^a^	2.49 ± 0.43^a^	2.18 ± 0.73^b^	2.01 ± 0.71^b^	2.58 ± 0.79^a^
HDL(mmol/l)	1.22 ± 0.096	0.7 ± 0.4^a^	0.58 ± 0.19^a^	0.64 ± 0.32^a^	0.89 ± 0.32	1.25 ± 0.26^b^
LDL (mmol/l)	0.63 ± 0.26	2.31 ± 0.63^a^	0.6 ± 0.16^b^	0.59 ± 0.1^b^	0.63 ± 0.13^b^	0.55 ± 0.21^b^
TG (mmol/l)	1.02 ± 0.37	2.74 ± 0.41^a^	1.22 ± 0.3^b^	1.23 ± 0.58^b^	1.05 ± 0.59^b^	0.94 ± 0.32^b^
AST (U/l)	121.6 ± 14.01	161.4 ± 48.5	120 ± 11.74	155.4 ± 34	109.4 ± 28.48^b^	111.8 ± 45.04^b^
ALT (U/l)	31.2 ± 6.34	32.6 ± 7.73	28.2 ± 10.63	29.8 ± 11.6	28.2 ± 6.97	29.8 ± 8.04

Data are expressed as mean ± SEM (*n* = 6); ^a^ *p* < 0.05 vs control group; ^b^ *p* < 0.05 vs HFD group

We remarked that the four studied extracts have a significant corrective effect of LDL-C and TG levels compared to HFD group. In the case of HDL-C level, PJ and PSO treated groups presented a HDL-C level lower than that of HFD groups. This result indicated that dual PJ and PSO do not have a protective effect of HDL-C. Only PL exhibited a significant correction of HDL-C level estimated by 44% compared to HFD group.

For TC level, all pomegranate extracts decreased TC content when compared to HFD group but it is only significant in PP and PJ treated groups.

For the liver enzymes, we noted that HFD regime increased slightly AST level but did not affect ALT level compared to control groups. PP and PL extracts decreased significantly AST level against HFD group. This is can be explained by the hepato-protective effect of pomegranate peel and leaves. Some similar studies confirmed the same conclusion [30].

### Oxidative stress status

**Fig. 2:**
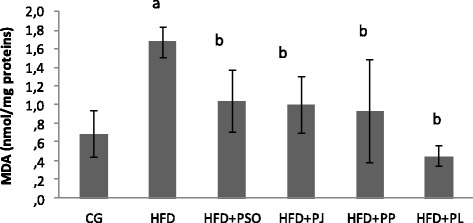
Effect of different pomegranate parts on MDA activity in HFD rat brain. Pomegranate Seeds Oil (PSO). Pomegranate Juice (PJ). Pomegranate Peel (PP) and Pomegranate Leaves (PL). Results are expressed as mean ± SEM. (*n* = 6). *P* < 0.05 was considered significant. ^a^ indicate significant difference compared to the values of CG. ^b^ indicate significant difference compared to the values of HFD groups

**Fig. 3:**
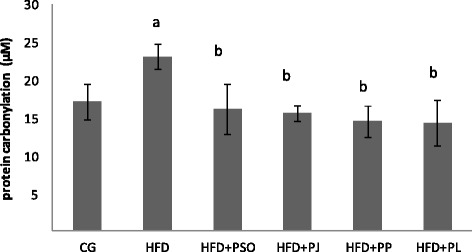
Effect of different pomegranate parts on protein carbonylation in HFD rat brain. Pomegranate Seeds Oil (PSO). Pomegranate Juice (PJ). Pomegranate Peel (PP) and Pomegranate Leaves (PL). Results are expressed as mean ± SEM. (*n* = 6). *P* < 0.05 was considered significant. ^a^ indicate significant difference compared to the values of CG. ^b^ indicate significant difference compared to the values of HFD groups

**Fig. 4:**
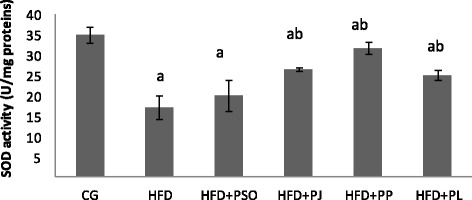
Effect of different pomegranate parts on SOD activity in HFD rat brain. Pomegranate Seeds Oil (PSO). Pomegranate Juice (PJ). Pomegranate Peel (PP) and Pomegranate Leaves (PL). Results are expressed as mean ± SEM. (*n* = 6). *P* < 0.05 was considered significant. ^a^ indicate significant difference compared to the values of CG. ^b^ indicate significant difference compared to the values of HFD groups

**Fig. 5:**
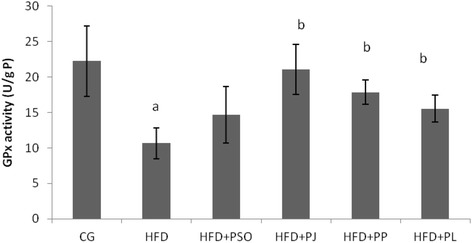
Effect of different pomegranate parts on GPx activity in HFD rat brain. Pomegranate Seeds Oil (PSO). Pomegranate Juice (PJ). Pomegranate Peel (PP) and Pomegranate Leaves (PL). Results are expressed as mean ± SEM. (*n* = 6). *P* < 0.05 was considered significant. ^a^ indicate significant difference compared to the values of CG. ^b^ indicate significant difference compared to the values of HFD groups

Figures 2, 3, 4 and 5 depicted the effect of HFD regime and pomegranate extracts consumption in oxidative stress status in rat brain. MDA is a biomarker of oxidative stress and most studied in elevation of polyunsaturated fatty acid peroxidation [43] .As shown in Fig. 2, brain MDA level was 60% higher in HFD group than that of control group. All pomegranate extracts administration decreased significantly the level of MDA by 70% and 47% in respectively PL and PP treated groups and by 40% in dual PSO and PJ treated groups.

Furthermore, similar results were found for PC. In fact, as illustrated in Fig. 3, HFD regime increased PC level by 26% in rat brain of HFD group when compared to control group. However, the administration of pomegranate extracts restored PC level by reduction of 30% in all treated groups.

Brain SOD and GPx levels were affected also by HFD regime. In fact, as illustrated in Figs. 4 and 5, HFD group exhibited lower levels of SOD and GPx compared to that of control group. The reduction was estimated by 50%. Consumption of PJ, PP and PL improved significantly levels of both enzymes compared to HFD group. However, No corrective effect was recorded to PSO.

## Discussion

Our study which is the first study about the neuroprotective effect of pomegranate extracts on obese animal, shows that different pomegranate parts: leaves, juice, peel and seeds oil not only have a significant anti-obesity effect but also a neuroprotective potential on the development of diet-induced obesity.

AChE is an acetylcholine hydrolyzing enzyme that is responsible for the termination of cholinergic response. An increased AChE activity in brain provoked memory deficits as well as oxidative stress [44, 45]. In present study, HFD regime induced increase in AchE activity. This observation is confirmed in previous experiments [42, 46]. However, long-term of pomegranate extracts consumption was found to reduce AChE activity. AChE activity depends largely on the membrane characteristics, since the enzyme is membrane bound. Increased activity of AChE is attributed to Ca2+ influx-mediated oxidative stress caused by Amyloid beta peptides. The mechanism of anticholinesterase activity of pomegranate may be due to antioxidant power of its bioactive compounds such as flavonoids. In fact, Ishige et al., found three distinct mechanisms of protection of neural cell from oxidative insults by flavonoids. These include preventing the influx of Ca2+ despite high levels of ROS, increasing intracellular GSH and directly lowering levels of ROS [47].

In addition, our results showed that oral administration of PJ and PP not only decreased body weight and improved lipids serum levels of HFD rats but also decreased significantly brain weight and attenuate brain oxidative stress by downregulating MDA and PC levels and upregulating SOD and GPx levels. Really, the antioxidant properties of pomegranate extracts have been well documented, which include free radical scavenging and inhibition of LPO as well as enhancement of antioxidant status and neuroprotection [48, 49]. Braidy et al. 2013 reported that PJ attenuate cytotoxicity and restore the redox imbalance caused by toxin treatment in Human Primary Neurons [18]. Subash et al. 2014 reported that diet supplemented by 4% of pomegranate extract attenuate oxidative damage in cortex and hippocampus by decrease in lipoperoxidation and protein carbonylation and restoration in the antioxidant enzymes level such as SOD, catalase (CAT), Glutathione peroxidase (GPx) and Glutathione (GSH) and inhibition of AchE activity [50]. Same findings were recorded also in others studies [51–53]. The neuroprotective effect of pomegranate peel and juice can be explained by their richness in bioactive compounds such as anthocyanins and tannins. In fact, Punicalagin (PG), a major compound in PJ and PP and widely known by power antioxidant activity was reported to have a potent neuroprotective effect against cerebral ischemia reperfusion induced oxidative brain injury in rats [54]. One of the possible mechanisms of neuroprotective effect of punicalagin was an improvement of Na+/K+ ATPase activity, an integral membrane protein which maintains electrolyte and fluid balance in cells, organs, and the whole body [54]. ATPase is known to be highly susceptible to changes in the membrane lipids, which may be further attributed to the progressive increase in the Lipid peroxidation (LPO) products especially MDA levels [55, 56]. Modification in Na + K + −ATPase activity may induce neuronal death with features of both apoptosis and necrosis [57]. So a decrease in MDA level and membrane LPO by punicalagin can improve Na+/K+ ATPase activity which drive the ion pumps to maintain neuron depolarization and thereby cellular integrity [54].

PSO is rich in polyunsaturated fatty acids, such as punicic acid, which is known to have hypolipidemic effect by suppression of TG synthesis in liver, through inhibition of fatty acid synthase [51]. In this study, PSO reduced significantly TG and LDL-C level but it hasn’t any effect against HDL-C. Concerning brain parameters, results show that PSO inhibit significantly and in dose dependant manner in vitro human blood AchE activity, decreased slightly BchE activity in brain homogenate, and improved significantly brain oxidative status without any significant corrective effect on brain weight. The neuroprotective effect of PSO was confirmed previously following administration of Nano-PSO, a nanodroplet formulation of PSO, to mice suffering from neurodegenerative disease [58].

## Conclusion

This study demonstrates that chronic intake of pomegranate extracts prevents against HFD complications such as hyperlipidemia and cerebral oxidative stress. In fact, the administration pomegranate juice, peel and seeds oil to HFD-fed rat deceased body weight, restored serum lipid levels, and attenuated oxidative stress in brain. All extracts showed significant inhibitory effect against human blood AchE activity in vitro study. An inhibitory effect against BChE in brain homogenate was also recorded after administration of different pomegranate extracts but this inhibition was statistically not significant. Taking into consideration all the demonstrated therapeutic effects of different pomegranate parts, this study underscores the neuroprotective potential of pomegranate fruits and encourages for further research to determine the bioactive mechanism.

## Abbreviations

AchE: Acetylcholinesterace Enzyme
ALT: Alanine Aminotransferase
AST: Aspartate Aminotransferase
BchE: Butyrylcholinesterace Enzyme
BMI: Body Mass Index
BSA: Bovine serum albumin
BW: Body weight
CAT: Catalase
CG: Control group
DNPH: 2, 4, dinitrophenylhydrazine
DNTB: 5.5 5, 5′-Dithiobis 2-nitrobenzoic acid
GPx: Glutathione peroxidase
GSH: Glutathione
HDL-C: High density lipoprotein cholesterol
HFD: High fat high fructose diet
LDL-C: Low density lipoprotein cholesterol
MDA: Malondialdehyde
NBT: Nitroblue tetrazolum
PC: Protein carbonylation
PJ: Pomegranate juice
PL: Pomegranate leaves
PP: Pomegranate peel
PSO: Pomegranate seeds oil
PTCI: Propionylthiocholine iodide
ROS: Reactive Oxygen Species (ROS)
SOD: Superoxide dismutase
TBARS: Thiobarbituric acid reactive substances
TC: Total cholesterol
TCA: Trichloroacetic acid
TG: Total triglycerides
